# Synthesis, crystal structure, Hirshfeld surface analysis and energy framework calculations of *trans*-3,7,9,9-tetra­methyl-10-(prop-2-yn-1-yl)-1,2,3,4,4a,9,9a,10-octa­hydro­acridine

**DOI:** 10.1107/S2056989021001183

**Published:** 2021-02-05

**Authors:** Mauricio Acelas, Analio Dugarte-Dugarte, Arnold R. Romero Bohórquez, José Antonio Henao, José Miguel Delgado, Graciela Díaz de Delgado

**Affiliations:** a Grupo de Investigación en Compuestos Orgánicos de Interés Medicinal (CODEIM), Parque Tecnológico Guatiguará, Universidad Industrial de Santander, Piedecuesta, Colombia; b Laboratorio de Cristalografía-LNDRX, Departamento de Química, Facultad de Ciencias, Universidad de los Andes, Mérida, Venezuela; c Grupo de Investigación en Química Estructural (GIQUE), Escuela de Química, Facultad de Ciencias, Universidad Industrial de Santander, Bucaramanga, Colombia

**Keywords:** octa­hydro­acridine, Povarov reaction, Hirshfeld surface, energy frameworks, DFT calculations, spectroscopic characterization, crystal structure

## Abstract

The title heterocyclic compound, C_20_H_27_N, has been prepared in good yield (72%) *via* a BiCl_3_-catalyzed cationic Povarov reaction between *N*-propargyl-4-methyl­aniline and (±)-citronellal. The X-ray single-crystal study indicates that the structure consists of mol­ecules connected by C—H⋯π contacts to produce chains, which pack in a sandwich–herringbone fashion along the *b*-axis direction. Hirshfeld surface analysis indicates that H⋯H inter­actions dominate by contributing 79.1% to the total surface. Energy frameworks and DFT calculations indicate a major contribution of dispersive forces to the total inter­action energy.

## Abstract 2

In the crystal of the new title octa­hydro­acridine, the mol­ecules are connected by C—H⋯π inter­actions, forming chains propagating along the *b*-axis direction that stack in a sandwich–herringbone arrangement.

## Chemical context   

The octa­hydro­acridine (OHA) scaffold is a synthetic nitro­gen heterocycle of significant importance in the fields of organic and medicinal chemistry. Its biological and pharmacological potential applications have been demonstrated over past decades (Ermolaeva *et al.*, 1968 ▸; Del Giudice *et al.*, 1997 ▸; Ulus *et al.*, 2016 ▸). The assembly of the OHA motif has been achieved by synthetic routes involving classic Beckman rearrangement (Sakane *et al.*, 1983 ▸), intra­molecular Friedel–Crafts acid-mediated cyclization (Kouznetsov *et al.*, 2000 ▸) and multicomponent amino­cyclization reactions (Selvaraj & Assiri, 2019 ▸). Noticeably, other approaches such as the organo­catalytic aza-Michael/aldol (Li *et al.*, 2018 ▸) and the Povarov reactions (Wu & Wang, 2014 ▸) have emerged as powerful tactics to control stereochemical features around the OHA core involving, for example, the selective insertion of multiple stereocenters. Moreover, the cationic version of the above mentioned Povarov reaction can be used to exploit natural sources of chemicals, demonstrating that citronellal, the major component of citronella essential oil, provides an expedite and diastereoselective alternative towards *N*-substituted OHAs (Acelas *et al.*, 2017 ▸).

The direct *N*-insertion of reactive groups, such as the propargyl fragment, *via* cationic Povarov reaction, enables access to multiple mol­ecular hybrids. This rational and relevant synthetic strategy prompts advantages such as broadening the pharmacological spectrum of several heterocycles and the enhancement in the therapeutic potential for specific diseases (Müller-Schiffmann *et al.*, 2012 ▸; Güiza *et al.*, 2019 ▸). Thus, some examples including OHA-isoxazole and OHA-1,2,3-triazole mol­ecular hybrids have already been described (Acelas *et al.*, 2019 ▸).

Despite the potential applications as pharmacological models, only a few examples of OHA crystal structures have been reported. It must be mentioned that the structural features obtained from the crystallographic data have been of the utmost importance and have served to accurately describe the stereochemical preference of different OHA synthesis pathways (Li *et al.*, 2018 ▸; Zaliznaya *et al.*, 2016 ▸), illustrate mol­ecular conformations (Fröhlich *et al.*, 1994 ▸; Gan *et al.*, 2000 ▸), and establish the effect of the reagent source (citronellal *vs* citronella essential oil) in the OHA crystal structure obtained *via* cationic Povarov reaction (Acelas *et al.*, 2020 ▸).
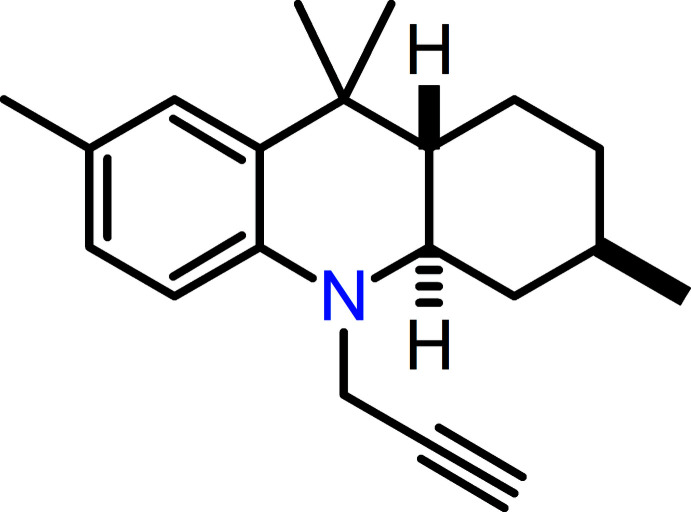


Herein, the synthesis, spectroscopic characterization, crystal structure and theoretical study of a new octa­hydro­acridine, *trans*-*N*-propargyl-3,7,9,9-tetra­methyl-1,2,3,4,4a,9,9a,10-octa­hydro­acridine, C_20_H_27_N, are described.

## Structural commentary   

**Figure 1:**
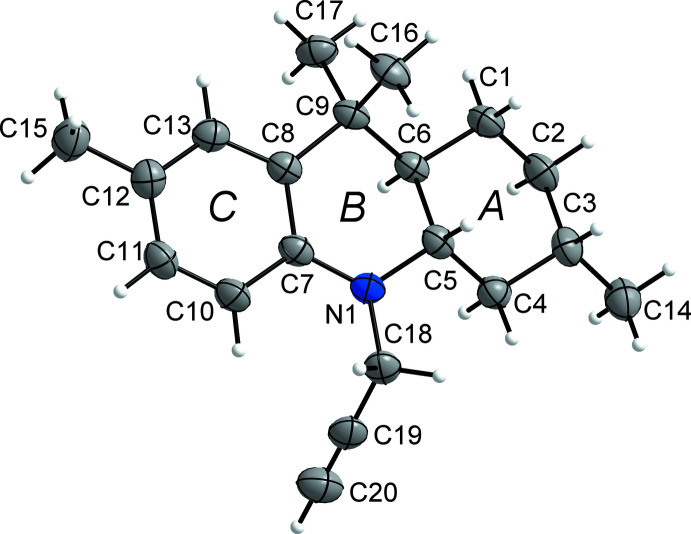
The mol­ecular structure of **3** with the atom- and ring-labeling scheme. Ellipsoids are drawn at the 30% level of probability.

Fig. 1 ▸ shows the mol­ecular structure of the title compound (**3**) with the atom- and ring-labeling scheme. The compound crystallizes with one mol­ecule in the asymmetric unit in space group *P*2_1_2_1_2_1_. The analysis of ring geometry parameters with *PLATON* (Spek, 2020 ▸) indicates that ring *A* has a chair conformation. Atoms N1 and C9 are equatorial with respect to atoms C5 and C6, respectively. This leads to a *trans* configuration for the fusion of rings *A* and *B*. The angle N1—C18—C19 is 112.97 (15)°, which can be correlated to the angle between the N—C≡C unit and the plane containing rings *B* and *C* (Fig. 1 ▸). A calculation carried out with *Mercury* (Macrae *et al.*, 2020 ▸) for the related hydro­quinoline structures discussed in the *Database survey* section below indicates this value ranges from 110.76 to 113.53° with a mean value of 112.45°. The C≡C bond length in compound **3** is 1.168 (3) Å, in excellent agreement with the mean value observed in related structures (1.169 Å). The relative stereochemistries of atoms C3, C5 and C6 in the crystal studied are *S*, *S* and *R*, respectively.

## Supra­molecular features   

**Figure 2:**
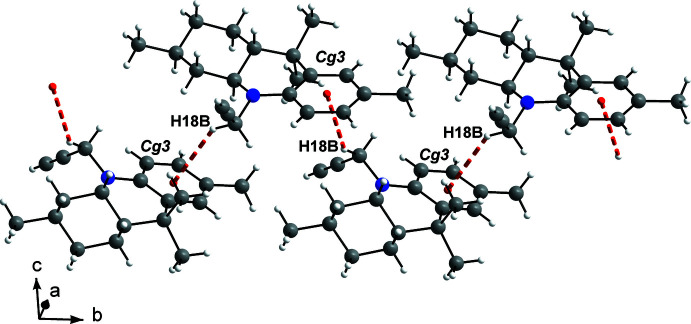
The packing of **3** showing chains of mol­ecules connected by C—H⋯π inter­actions along the *b*-axis direction.

**Figure 3:**
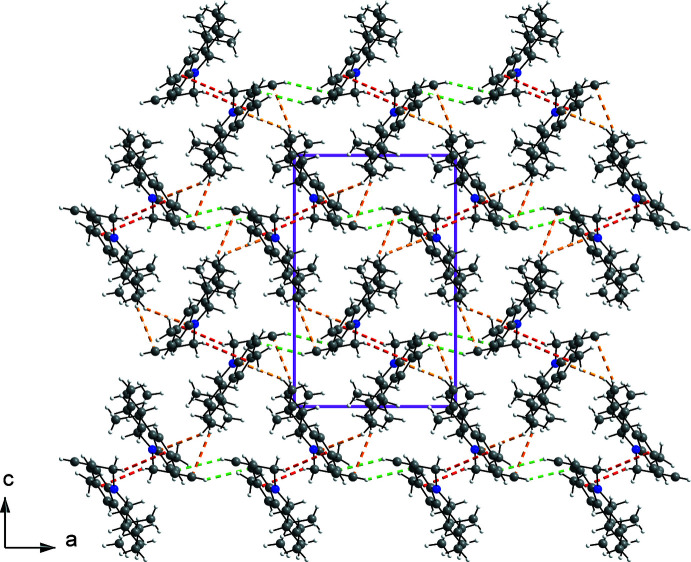
The packing arrangement viewed down [010]. Some short contacts are shown with dashed lines: C—H⋯*Cg*3 in red and C—H⋯C in orange and green.

In the crystal, the mol­ecules of **3** inter­act *via* C—H⋯π contacts between the –CH—C≡C grouping of a mol­ecule and the centroid (*Cg*3) of ring *C* of a mol­ecule related by symmetry operation (i) [1 − *x*, 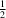 + *y*, 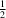 − *z* (2_1_ screw axis along *b*)] to form helical chains propagating along the *b*-axis direction (Fig. 2 ▸). The H⋯*Cg*3 distance is 2.98 Å and the C—H⋯*Cg*3 angle is 146°. The chains form columns, which inter­act *via* weak C—H⋯C contacts and van der Waals inter­actions. Some of these contacts are shown in Fig. 3 ▸. For example, C11⋯H20^ii^ contacts (3.00 Å, C10—C11⋯H20 = 104°, shown in green) link the columns along the *a*-axis direction. Additional inter­actions involving C7 and C20 (shown in orange) with atoms H2*B* and H1*A*, respectively, of a mol­ecule related by symmetry operation (iii) (−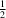 + *x*, 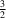 − *y*, 1 − *z*), connect the columns along the *c*-axis direction (C7⋯H2*B* = 3.05 Å, C8—C7⋯H2*B* = 100°; C20⋯H1*A* = 3.03 Å, C19—C20⋯H1*A* = 100°). The columns pack in a basket-weave tiling fashion (Fig. 3 ▸), also described as a sandwich–herringbone motif (Loots & Barbour, 2012 ▸).

## Hirshfeld surface analysis and energy framework calculations   

**Figure 4:**
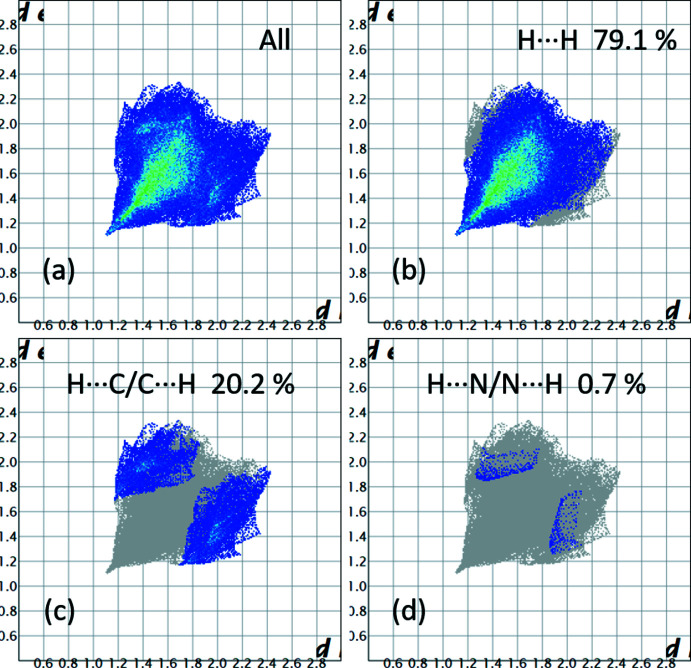
Fingerprint plots for the *d*_norm_ parameter mapped onto the Hirshfeld surface for **3**.

**Figure 5:**
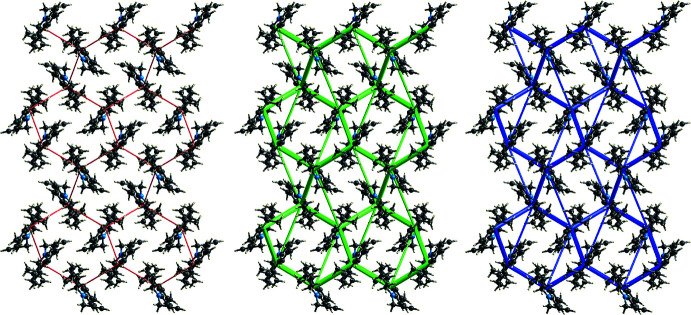
Energy frameworks calculated for compound **3** viewed down [010] represented within 2 × 2 × 2 unit cells. The radii of the cylinders were scaled to 80 arbitrary units with a cut-off value of 10 kJ mol^−1^. *E*_ele_ *, E*_dis_, and *E*_tot_ are represented (left to right) in red, green, and blue, respectively.

The *d*_norm_ parameter was mapped over the Hirshfeld surface (Spackman & Jayatilaka, 2009 ▸) and fingerprint plots were produced with *CrystalExplorer17.5* (Turner *et al.*, 2017 ▸) as shown in Fig. 4 ▸. The plots indicate the structure is dominated by H⋯H contacts, which account for 79.1% of the total inter­actions. The H⋯C/C⋯H inter­actions contribute 20.2% while the H⋯N/N⋯H contacts account for only 0.7%. Energy framework calculations resulted, as expected, in a major contribution of dispersive energies to the total energy, as seen in Fig. 5 ▸. The topology of the energy frameworks resemble a tilted honeycomb arrangement when viewed down the *b*-axis direction. Fig. S1 (supporting information) shows the Hirshfeld surface of a central mol­ecule and the neighboring mol­ecules in close contact. A comparison of *d*_norm_, shape index and curvedness mapped onto the Hirshfeld surface is presented in Fig. S2. The absence of adjacent red and blue triangular motifs in the shape index and of flat areas in the curvedness plots agrees with the absence of π–π inter­actions in the structure.

## Theoretical study   

The results of the calculations (Stewart, 2008 ▸, 2016 ▸, 2018 ▸) carried out with the PM6 (Stewart, 2007 ▸), PM7 (Stewart, 2013 ▸) and PM6-DH2 (Korth *et al.*, 2010 ▸) methods for compound **3** are presented in Tables S1 to S4 of the supporting information. The best results were obtained with PM7. The excellent agreement between the experimental crystal structure and the energy-minimized structure is noted by the low RMSD (0.023 Å) as shown in Table S5. Fig. S3 shows the agreement between the experimental and the energy-minimized structure. The optimized unit-cell parameters are very close to the values obtained in the single-crystal experiments. The unsigned mean error deviation UME(*a*,*b*,*c*,α,β,γ) is 0.453. The value obtained for the density and the unit-cell volume confirmed the good accuracy of the results. The greater contribution of the dispersive forces to the heat of formation was expected after the crystallochemical and Hirshfeld analyses. Energy-related parameters calculated are summarized in Table S6.

## Database survey   

A search of the Cambridge Structural Database (CSD, version 5.41, November 2019, update of 2 May 2020; Groom *et al.*, 2016 ▸) using as search criterion the *N*-propargyl-octa­hydro­acridine moiety without any substituents, did not result in structures of this type. A further search for *N*-propargyl hydro­quinolines resulted in only eight related compounds: refcodes FORCAT (Filali Baba *et al.*, 2019 ▸), KEPRUU (Dixit *et al.*, 2012 ▸), POWVIJ (Hayani *et al.*, 2019 ▸), UQODUA (Suzuki *et al.*, 2010 ▸), UROJUI and UROKAP (Shakoori *et al.*, 2013 ▸), WIYCIR (Suzuki *et al.*, 2008 ▸) and XILYUP (Filali Baba *et al.*, 2017 ▸). Of these compounds, KEPRUU is perhaps the most closely related to the compound reported here. However, it contains substituents (F, Cl, oxo, and ethyl carboxyl­ate), which would render a richer display of inter­molecular inter­actions.

## Synthesis and crystallization   

All reagents were purchased from Merck and used without additional purification. *N*-Propargyl-4-methyl­aniline was prepared (see scheme below) according to a previously reported procedure (Sakai *et al.*, 2017 ▸). TLC aluminum sheets PF254 from Merck were employed to monitor the reaction progress. Column chromatography was performed using silica gel (60–120 mesh). The melting point (uncorrected) was determined using a Fisher–Johns melting point apparatus. A solution of *N*-propargyl-4-methyl­aniline (**1**, 0.449 g, 3.09 mmol) and (±)-citronellal (**2**, 0.477 g, 3.09 mmol) in 5 ml of aceto­nitrile was poured into a 50 ml round-bottom flask and stirred at room temperature for 10 min; the catalyst BiCl_3_ (0.097 g, 10 mol %) was then added to the mixture. After 6 h of reaction as indicated by TLC, 15 ml of a saturated NaHCO_3_ aqueous solution was added and the crude product was extracted with ethyl acetate (20 ml × 3) and dried over Na_2_SO_4_. The *cis*/*trans* octa­hydro­acridine mixture (1:9 determined by GC) was purified using petroleum ether (b.p. 313–333 K) as eluent. Further recrystallization from petroleum ether solution gave only the *trans* product (**3**) (see reaction scheme). Yellow solid, m.p. 347–348 K. (0.625 g) 72% yield. Analysis calculated for C_20_H_27_N: C, 85.35; H, 9.67; N, 4.98%. Found: C, 85.87; H, 9.52; N, 5.05%.
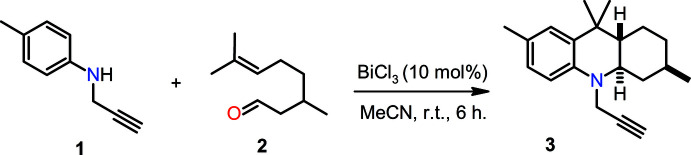


## X-ray powder diffraction   

**Figure 6:**
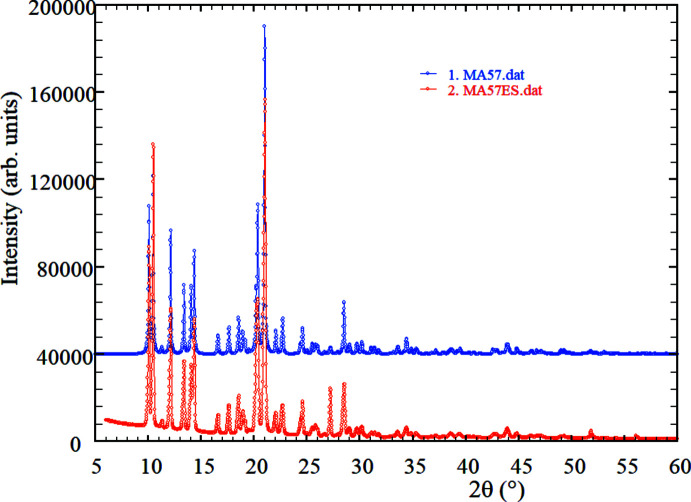
X-ray powder diffraction patterns of compound **3**. Experimental (bottom, red) and simulated from single-crystal data (top, blue).

The powder pattern recorded was indexed on a primitive ortho­rhom­bic unit cell with *a* = 15.650 (3), *b* = 10.626 (2), *c* = 10.054 (1) Å, *V* = 1672.1 (2) Å^3^, using *DICVOL14* (Louër & Boultif, 2014 ▸), in excellent agreement with the unit-cell parameters obtained from the single-crystal data collection. All 61 diffraction maxima registered were indexed with good figures-of-merit: *M*_20_ = 23.8 (de Wolff *et al.*, 1968 ▸) and *F*_20_ = 63.4 (0.0096, 33) (Smith & Snyder, 1979 ▸). Since the powder diffraction pattern of this material has not been previously reported, the data have been sent to the Inter­national Center for Diffraction Data (ICDD) for its inclusion in the Powder Diffraction File (Gates-Rector & Blanton, 2019 ▸). As can be seen in Fig. 6 ▸, the pattern recorded looks almost identical to the pattern calculated using the structural data obtained from the single-crystal structure-determination process. The absence of impurity lines in the powder diffraction pattern recorded confirms that the synthetic route employed produced selectively the desired compound.

## Spectroscopic characterization   

**Figure 7:**
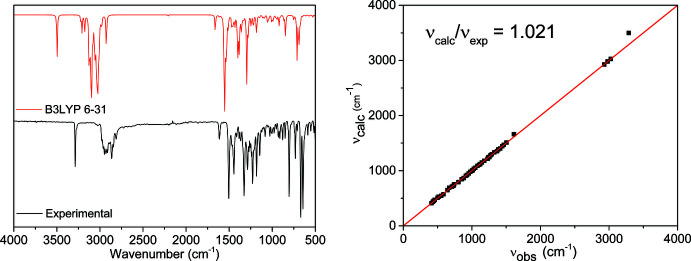
Experimental and calculated (B3LYP 6–31) IR spectra of compound **3** and Correlation between calculated ν_calc_ and observed ν_obs_ frequencies.

The results are summarized in Table 1 ▸. The ATR–FTIR spectrum (Fig. 7 ▸) shows the absence of the N—H and C=O stretch bands around 3350 and 1740 cm^−1^, indicating complete reaction of the aniline and citronellal precursors, respectively. The assignment and confirmation of fundamental vibrational modes was performed by direct correlation after geometry optimization and vibrational frequency calculations (Neugebauer & Hess, 2003 ▸) carried out with *Gaussian 09* (Frisch *et al.*, 2009 ▸) using the B3LYP/6-31 basis set (Hehre *et al.*, 1972 ▸; Petersson & Al-Laham, 1991 ▸). High accuracy is observed for vibrational frequencies in the 1500–500 cm^−1^ range (Fig. 7 ▸). However, for vibrations above 1500 cm^−1^, an increase in the error between the observed and calculated frequencies is more noticeable, as previously described for other DFT vibrational studies (Matsuura & Yoshida, 2006 ▸). A sharp and strong signal at 3286 cm^−1^, attributed to the C≡CH stretch, serves as evidence of the propargyl *N*-substituent group presence. An additional absorption band at 3024 cm^−1^ is observed and corresponds to the aromatic C—H stretch in the OHA mol­ecule. Absorptions at 1614 and 1504 cm^−1^ are attributed to the C=C aromatic stretch and the band at 1182 cm^−1^ is assigned to the C—N stretch vibration.

**Figure 8:**
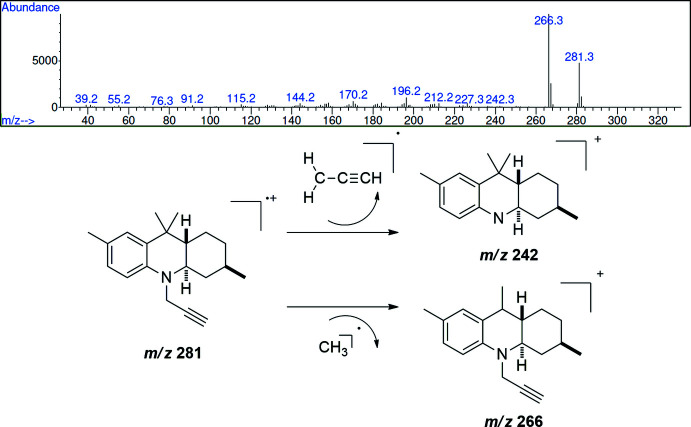
EI (70 eV) mass spectrum of **3** and main fragmentation pattern observed in the MS spectrum.

The mass spectrum (EI, 70 eV) for the title compound is depicted in Fig. 8 ▸. The mol­ecular ion at 281.3 *m*/*z* is observed with a relative intensity of 47% and it is in accordance with the mol­ecular formula C_20_H_27_N. Peaks at 266 and 242 *m*/*z* are attributed to fragmentations involving the loss of a methyl group inducing the formation of a very stable benzylic tertiary cation and the loss of the propargyl fragment, respectively.

**Figure 9:**
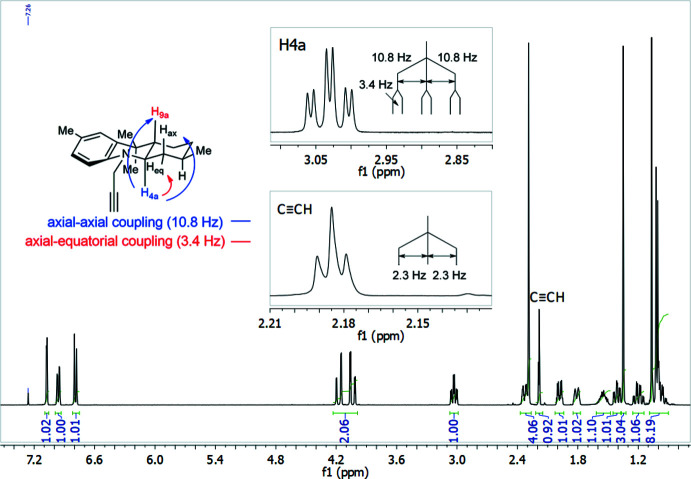
^1^H-NMR spectrum for **3**. The inserts emphasize the alkyne proton region and the assignment of a *trans*-fusion pattern of rings *A* and *B*.

**Figure 10:**
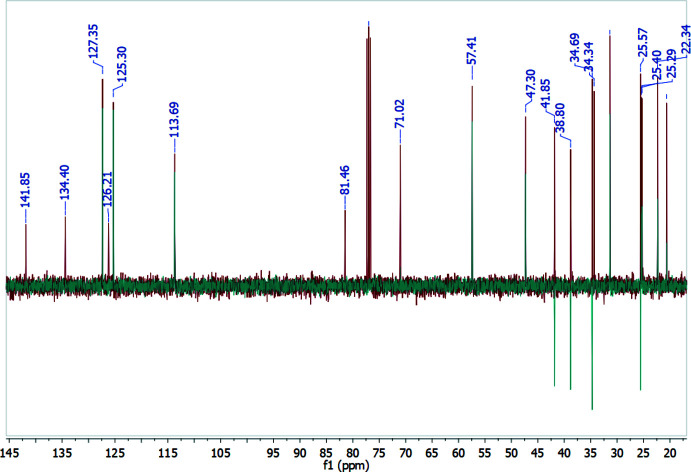
^13^C-NMR and DEPT-135 spectra.

The ^1^H-NMR spectrum (Fig. 9 ▸) shows the aromatic signals at downfield as doublets and doublet of doublets with their corresponding ^3^ *J* and ^4^ *J* values of 8.3 and 1.7 Hz, respectively. The methyl­enic protons of the propargyl moiety appear as two doublets of doublets at 4.04 and 4.17 ppm. Two singlets at 1.03 and 1.35 ppm correspond to the methyl groups bonded to *C-9*. The difference in their chemical shift values is the result of a distinct chemical environment due to a specific and non-inter­changeable mol­ecular conformation adopted by the OHA. The alkyne proton at 2.18 ppm appears as a triplet with ^4^ *J* = 2.3 Hz. The signal for the proton *H-4a* at 3.04 ppm (*td*, *J* = 10.8; 3.4 Hz) plays a key role in the spectroscopic determin­ation of the OHA stereochemistry. It suggests two pseudoaxial (10.8 Hz) and one pseudoequatorial (3.4 Hz) spin couplings which are characteristic of a *trans* geometry in fused rings, as observed in Fig. 9 ▸. All other aliphatic signals are located at high field, mainly as multiplets. The ^13^C-NMR spectrum, shown in Fig. 10 ▸, displays the characteristic signals for the propargyl group at 71.02 and 81.46 ppm. The signals for the methyl groups at *C-9* also have different chemical shift values, observed at 25.0 and 25.2 ppm. The determination of quaternary carbon atoms and differentiation between methyl, methyl­enic and methynic groups was achieved using the DEPT-135 spectrum (Fig. 10 ▸).

## Refinement   

Crystal data, data collection and structure refinement details are summarized in Table 2 ▸. Hydrogen atoms were identified in the difference-Fourier map but were included in geometrically calculated positions (C—H = 0.93–0.98 Å) and refined as riding with *U*_iso_(H) = 1.2–1.5*U*_eq_(C).

## Supplementary Material

Crystal structure: contains datablock(s) I. DOI: 10.1107/S2056989021001183/hb7966sup1.cif

Structure factors: contains datablock(s) I. DOI: 10.1107/S2056989021001183/hb7966Isup2.hkl

Supporting information file. DOI: 10.1107/S2056989021001183/hb7966Isup3.mol

Tables of Theoretical study results. DOI: 10.1107/S2056989021001183/hb7966sup4.pdf

Supporting information file. DOI: 10.1107/S2056989021001183/hb7966Isup5.cml

CCDC reference: 2004267

Additional supporting information:  crystallographic information; 3D view; checkCIF report

## Figures and Tables

**Table 1:** Analytical data for **3**: ATR–FTIR, IE–MS, ^1^H-NMR, ^13^C-NMR

**ATR–FTIR (cm^−1^)**		
3286 ν (C≡CH)	2947 ν (CH)	2929 ν (CH)
2864 ν (CH)	1504 ν (C=C_arom_)	1182 ν (C—N)
		
**MS (EI), *m*/*z* (%)**		
281.3 (*M* ^·+^; 47)	267.3 (26)	266.3 (100)
		
**^1^H-NMR (CDCl_3_, 400 MHz, ppm)**		
δ_H_ 0.91–1.05 (*m*, 2H_2,4_)	1.01 (*d*, *J* = 6.7 Hz, 3H_3-Me_)	1.07 (*s*, 3H_9-Me_)
1.14–1.26 (*m*, 1H_1_)	1.35 (*s*, 3H_9-Me_)	1.41 (*td*, *J* = 11.4, 3.2 Hz, 1H_9a_)
1.48–1.62 (*m*, 1H_3_)	1.77–1.85 (*m*, 1H_2_)	1.95–2.02 (*m*, 1H_1_)
2.18 (*t*, *J* = 2.3 Hz, 1H_1-Proparg­yl_)	2.29 (*s*, 3H_7-Me_)	2.30–2.36 (*m*, 1H_4_)
3.03 (*td*, *J* = 10.8, 3.5 Hz, 1H_4a_)	4.04 (*dd*, *J* = 18.4, 2.3 Hz, 1H_CH2-Proparg­yl_)	4.17 (*dd*, *J* = 18.4, 2.3 Hz, 1H_CH2-Proparg­yl_)
6.79 (*d*, *J* = 8.3 Hz, 1H_5_)	6.96 (*ddd*, *J* = 8.3, 1.7, 0.6 Hz, 1H_6_)	7.08 (*d*, *J* = 1.6 Hz, 1H_8_)
		
**^13^C-NMR (CDCl_3_, 100 MHz, ppm)**		
δ_C_ 20.64_(7-Me)_	22.34_(3-Me)_	25.29_(9-Me)_
25.41_(9-Me)_	25.57_(4)_	31.36_(3)_
34.34_(9)_	34.69_(2)_	38.80_(CH2-Proparg­yl)_
41.85_(1)_	47.30_(9a)_	57.41_(4a)_
71.02_(1-Proparg­yl)_	81.46_(2-Proparg­yl)_	113.70_(5)_
125.30_(6)_	126.21_(7)_	127.35_(8)_
134.40_(8a)_	141.85_(10*a*)_	

Signals were designated as: *s*, singlet; *d*, doublet; *dd*, doublet of doublets; *ddd*, doublet of doublets of doublets; *t*, triplet; *td*, triplet of doublets; *q*, quartet; *m*, multiplet; *br.*, broad.

**Table 2:** Experimental details

Crystal data	
Chemical formula	C_20_H_27_N
*M*_r_	281.42
Crystal system, space group	Orthorhombic, *P*2_1_2_1_2_1_
Temperature (K)	293
*a*, *b*, *c* (Å)	10.05103 (9), 10.62943 (11), 15.64759 (16)
*V* (Å^3^)	1671.74 (3)
*Z*	4
Radiation type	Cu *K*α
μ (mm^−1^)	0.48
Crystal size (mm)	0.48 × 0.33 × 0.29

Data collection	
Diffractometer	Rigaku Pilatus 200K
Absorption correction	Multi-scan (*CrysAlis PRO*; Rigaku OD, 2019 ▸)
*T*_min_, *T*_max_	0.573, 1.000
No. of measured, independent and observed [*I* > 2σ(*I*)] reflections	6660, 3181, 3160
*R*_int_	0.013
(sin θ/λ)_max_ (Å^−1^)	0.624

Refinement	
*R*[*F* ^2^ > 2σ(*F* ^2^)], *wR*(*F* ^2^), *S*	0.039, 0.107, 1.11
No. of reflections	3181
No. of parameters	195
H-atom treatment	H-atom parameters constrained
Δρ_max_, Δρ_min_ (e Å^−3^)	0.15, −0.19
Absolute structure	Flack *x* determined using 1267 quotients [(*I* ^+^)−(*I* ^−^)]/[(*I* ^+^)+(*I* ^−^)] (Parsons *et al.*, 2013 ▸)
Absolute structure parameter	0.3 (2)

Computer programs: *CrystalClear-SM Expert* (Rigaku/MSC, 2015 ▸), *CrysAlis PRO* (Rigaku OD, 2019 ▸), *SHELXT2014/5* (Sheldrick, 2015*a* ▸), *SHELXL2018/3* (Sheldrick, 2015*b* ▸), *DIAMOND* (Brandenburg, 1999 ▸), *OLEX2* (Dolomanov *et al.*, 2009 ▸), *enCIFer* (Allen *et al.*, 2004 ▸) and *publCIF* (Westrip, 2010 ▸).

## supplementary crystallographic information

### Crystal data

**Table:**

C_20_H_27_N	*D*_x_ = 1.118 Mg m^−^^3^
*M**_r_* = 281.42	Melting point: 367 K
Orthorhombic, *P*2_1_2_1_2_1_	Cu *K*α radiation, λ = 1.54184 Å
*a* = 10.05103 (9) Å	Cell parameters from 6301 reflections
*b* = 10.62943 (11) Å	θ = 2.8–74.2°
*c* = 15.64759 (16) Å	µ = 0.48 mm^−^^1^
*V* = 1671.74 (3) Å^3^	*T* = 293 K
*Z* = 4	Block, colorless
*F*(000) = 616	0.48 × 0.33 × 0.29 mm

### Data collection

**Table:**

Rigaku Pilatus 200K diffractometer	3181 independent reflections
Radiation source: fine-focus sealed X-ray tube, Enhance (Cu) X-ray Source	3160 reflections with *I* > 2σ(*I*)
Graphite monochromator	*R*_int_ = 0.013
Detector resolution: 5.8140 pixels mm^-1^	θ_max_ = 74.2°, θ_min_ = 5.7°
profile data from ω–scans	*h* = −9→11
Absorption correction: multi-scan (CrysAlisPRO; Rigaku OD, 2019)	*k* = −11→13
*T*_min_ = 0.573, *T*_max_ = 1.000	*l* = −19→19
6660 measured reflections	

### Refinement

**Table:**

Refinement on *F*^2^	Hydrogen site location: inferred from neighbouring sites
Least-squares matrix: full	H-atom parameters constrained
*R*[*F*^2^ > 2σ(*F*^2^)] = 0.039	*w* = 1/[σ^2^(*F*_o_^2^) + (0.0679*P*)^2^ + 0.0783*P*] where *P* = (*F*_o_^2^ + 2*F*_c_^2^)/3
*wR*(*F*^2^) = 0.107	(Δ/σ)_max_ < 0.001
*S* = 1.11	Δρ_max_ = 0.15 e Å^−^^3^
3181 reflections	Δρ_min_ = −0.19 e Å^−^^3^
195 parameters	Extinction correction: SHELXL2018/3 (Sheldrick, 2015b), Fc^*^=kFc[1+0.001xFc^2^λ^3^/sin(2θ)]^-1/4^
0 restraints	Extinction coefficient: 0.037 (4)
Primary atom site location: dual	Absolute structure: Flack *x* determined using 1267 quotients [(*I*^+^)-(*I*^-^)]/[(*I*^+^)+(*I*^-^)] (Parsons *et al.*, 2013)
Secondary atom site location: difference Fourier map	Absolute structure parameter: 0.3 (2)

### Special details

**Table:**

Experimental. *Characterization by X-ray powder diffraction*A small portion of the synthesized material, previously grounded in an agate mortar, was dusted on top of a flat plate low-background Si single crystal specimen holder. The powder diffraction pattern was registered at room temperature on a Bruker D8 ADVANCE diffractometer working in the Bragg-Brentano geometry using Cu *K*a radiation, operating at 40 kV and 30 mA, and equipped with a LynxEye position-sensitive detector. The pattern was recorded from 6.00 to 70.00° (2θ) in steps of 0.01526°, at 1 sec/step. The standard instrument settings (Ni filter of 0.02 mm, Soller slits of 2.5°, Divergence slit of 0.2 mm, scatter screen height of 3 mm) were employed.*Characterization by ATR-FTIR, mass spectrometry, elemental analysis, and ^1^H- and ^13^C-NMR*The IR spectrum was recorded in the region from 4000 to 500 cm^-1^ on a Bruker Tensor 27 FTIR spectrophotometer coupled to a Bruker platinum ATR cell. Vibrational frequencies were calculated by the B3LYP method with a 6-31G basis set, as a strategy to correlate the experimental bands with their corresponding vibrational modes (Matsuura and Yoshida, 2006). The mass spectrum was recorded on a Hewlett Packard 5890a Series II Gas Chromatograph interfaced to an HP MS ChemStation Data System at 70 eV using a 60 m capillary column coated with HP-5 [5% phenylpoly(dimethylsiloxane)]. Elemental analysis was performed on a Thermo Scientific CHNS-O analyzer (Model Flash 2000) and the experimental values were within ± 0.4 of the theoretical values. NMR spectra (^1^H and ^13^C) were measured on a Bruker Ultrashield-400 spectrometer (400 MHz ^1^H NMR and 100 MHz ^13^C NMR), using CDCl_3_ as solvent and reference. *J* values are reported in Hz; chemical shifts are reported in ppm (δ) relative to the solvent peak (residual CHCl_3_ in CDCl_3_ at 7.26 ppm for protons). Signals were designated as: s, singlet; d, doublet; dd, doublet of doublets; ddd, doublet of doublets of doublets; t, triplet; td, triplet of doublets; q, quartet; m, multiplet; br., broad.*Geometry and energy optimization*Semi-empirical quantum chemistry calculations were performed to evaluate the crystalline structure determined using single crystal X-ray diffraction techniques. The calculations were carried out using the treatment of periodic boundary conditions (Stewart, 2008) implemented in the MOPAC2016 package (Stewart, 2016). A laptop equipped with 1.60GHz Intel(R) Core(TM) i5-8250U CPU, 8Gb memory, and a Windows 10 operating system was used. To minimize border effects and obtain a full structure representation of the compound under study, the crystallographic unit cell was replicated l, m, and n times along the corresponding Cartesian axes. In each case, the keyword MERS = (l,m,n) was used, where l, m, and n could be either 1 or 2. Using the experimental crystal structure parameters, an input cluster of molecules for the compound was created using Mercury (Macrae *et al.*, 2020) and MAKPOL (Stewart, 2018). The studied clusters consisted of 768 and 384 atoms. The geometry was energy minimized using the L-BFGS-B function minimizer with the PM6 (Stewart, 2007), PM7 (Stewart, 2013) and PM6-DH2 (Korth *et al.*, 2010) methods, allowing the unit cell parameters and the atomic coordinates of all 768 and 384 atoms to vary in every case. The calculation was set to terminate when the gradient norm reached a value <10 Kcal mol-1 Å^-1^. The optimized atomic positions were visualized and compared to the experimental atomic coordinates using the Crystal Packing Similarity capability of Mercury.
Geometry. All esds (except the esd in the dihedral angle between two l.s. planes) are estimated using the full covariance matrix. The cell esds are taken into account individually in the estimation of esds in distances, angles and torsion angles; correlations between esds in cell parameters are only used when they are defined by crystal symmetry. An approximate (isotropic) treatment of cell esds is used for estimating esds involving l.s. planes.

### Fractional atomic coordinates and isotropic or equivalent isotropic displacement parameters (Å^2^)

**Table:**

	*x*	*y*	*z*	*U*_iso_*/*U*_eq_	
N1	0.38131 (16)	0.67573 (14)	0.32881 (9)	0.0587 (4)	
C1	0.5388 (2)	0.6967 (2)	0.54905 (12)	0.0651 (5)	
H1A	0.522689	0.649842	0.601246	0.078*	
H1B	0.631139	0.684239	0.533165	0.078*	
C2	0.5154 (2)	0.8351 (2)	0.56596 (13)	0.0698 (5)	
H2A	0.425363	0.847263	0.586694	0.084*	
H2B	0.576286	0.863906	0.609906	0.084*	
C3	0.53583 (17)	0.91189 (18)	0.48576 (13)	0.0620 (4)	
H3	0.628642	0.900743	0.468121	0.074*	
C4	0.44845 (17)	0.86058 (16)	0.41413 (12)	0.0566 (4)	
H4A	0.466096	0.907853	0.362330	0.068*	
H4B	0.355835	0.873562	0.429161	0.068*	
C5	0.47082 (15)	0.72069 (16)	0.39624 (10)	0.0508 (4)	
H5	0.562872	0.708887	0.377202	0.061*	
C6	0.44943 (15)	0.64426 (16)	0.47810 (9)	0.0484 (4)	
H6	0.357383	0.659273	0.496077	0.058*	
C7	0.34579 (16)	0.54953 (16)	0.32338 (9)	0.0517 (4)	
C8	0.38003 (15)	0.46188 (15)	0.38711 (9)	0.0492 (4)	
C9	0.46345 (16)	0.50046 (17)	0.46439 (9)	0.0513 (4)	
C10	0.27175 (19)	0.50620 (18)	0.25324 (11)	0.0621 (4)	
H10	0.246012	0.563166	0.211266	0.075*	
C11	0.23606 (19)	0.38138 (19)	0.24481 (12)	0.0648 (5)	
H11	0.188546	0.355866	0.196845	0.078*	
C12	0.26960 (18)	0.29373 (18)	0.30627 (12)	0.0620 (4)	
C13	0.34035 (17)	0.33742 (17)	0.37640 (11)	0.0579 (4)	
H13	0.362622	0.279976	0.418889	0.069*	
C14	0.5139 (2)	1.0515 (2)	0.49995 (16)	0.0780 (6)	
H14A	0.423655	1.065695	0.517634	0.117*	
H14B	0.530617	1.096137	0.447712	0.117*	
H14C	0.573460	1.081061	0.543498	0.117*	
C15	0.2335 (3)	0.1564 (2)	0.29657 (17)	0.0872 (7)	
H15A	0.222116	0.119406	0.352062	0.131*	
H15B	0.303458	0.113374	0.266677	0.131*	
H15C	0.152176	0.149164	0.264824	0.131*	
C16	0.60856 (18)	0.4612 (2)	0.44781 (13)	0.0689 (5)	
H16A	0.662066	0.482466	0.496540	0.103*	
H16B	0.641587	0.504656	0.398371	0.103*	
H16C	0.612541	0.372124	0.438170	0.103*	
C17	0.4133 (2)	0.43261 (19)	0.54534 (11)	0.0659 (5)	
H17A	0.463342	0.460816	0.593947	0.099*	
H17B	0.424506	0.343467	0.538626	0.099*	
H17C	0.320781	0.451316	0.553822	0.099*	
C18	0.37514 (19)	0.74991 (18)	0.24998 (11)	0.0614 (4)	
H18A	0.400066	0.696834	0.202166	0.074*	
H18B	0.439266	0.817919	0.253479	0.074*	
C19	0.2427 (2)	0.80308 (18)	0.23385 (11)	0.0646 (5)	
C20	0.1372 (3)	0.8443 (2)	0.21983 (15)	0.0835 (6)	
H20	0.053201	0.877163	0.208666	0.100*	

### Atomic displacement parameters (Å^2^)

**Table:**

	*U*^11^	*U*^22^	*U*^33^	*U*^12^	*U*^13^	*U*^23^
N1	0.0707 (9)	0.0631 (8)	0.0422 (7)	−0.0078 (7)	−0.0101 (6)	0.0074 (6)
C1	0.0652 (10)	0.0769 (11)	0.0531 (9)	−0.0001 (9)	−0.0168 (8)	0.0011 (8)
C2	0.0722 (11)	0.0804 (12)	0.0567 (10)	−0.0046 (9)	−0.0130 (8)	−0.0101 (9)
C3	0.0471 (8)	0.0699 (11)	0.0689 (10)	−0.0042 (8)	−0.0036 (8)	−0.0074 (9)
C4	0.0517 (8)	0.0632 (9)	0.0548 (8)	−0.0022 (7)	−0.0015 (7)	0.0025 (7)
C5	0.0431 (7)	0.0651 (9)	0.0443 (8)	−0.0003 (6)	0.0012 (6)	0.0006 (7)
C6	0.0403 (7)	0.0641 (9)	0.0407 (7)	0.0037 (6)	−0.0012 (5)	0.0012 (6)
C7	0.0500 (8)	0.0644 (9)	0.0405 (7)	0.0018 (7)	0.0005 (6)	0.0004 (6)
C8	0.0438 (7)	0.0622 (8)	0.0415 (7)	0.0039 (6)	0.0034 (6)	0.0000 (6)
C9	0.0485 (8)	0.0647 (9)	0.0406 (7)	0.0083 (7)	−0.0011 (6)	0.0022 (6)
C10	0.0706 (11)	0.0699 (10)	0.0458 (8)	−0.0005 (9)	−0.0104 (8)	0.0001 (7)
C11	0.0635 (10)	0.0775 (11)	0.0535 (9)	−0.0047 (8)	−0.0080 (8)	−0.0071 (8)
C12	0.0598 (9)	0.0662 (10)	0.0600 (9)	−0.0047 (8)	0.0047 (8)	−0.0057 (8)
C13	0.0581 (9)	0.0640 (9)	0.0515 (8)	0.0032 (7)	0.0038 (7)	0.0035 (7)
C14	0.0715 (12)	0.0732 (12)	0.0892 (15)	−0.0048 (10)	−0.0069 (10)	−0.0125 (11)
C15	0.1057 (17)	0.0729 (13)	0.0830 (14)	−0.0185 (12)	−0.0037 (13)	−0.0044 (11)
C16	0.0535 (9)	0.0854 (12)	0.0676 (10)	0.0171 (9)	−0.0066 (8)	−0.0088 (9)
C17	0.0826 (12)	0.0698 (10)	0.0454 (8)	0.0057 (9)	−0.0007 (8)	0.0085 (7)
C18	0.0699 (10)	0.0729 (10)	0.0415 (8)	−0.0074 (8)	0.0004 (7)	0.0087 (7)
C19	0.0803 (13)	0.0706 (10)	0.0429 (8)	−0.0053 (9)	−0.0014 (8)	0.0107 (7)
C20	0.0827 (15)	0.0995 (15)	0.0682 (13)	0.0113 (12)	−0.0027 (11)	0.0173 (12)

### Geometric parameters (Å, º)

**Table:**

N1—C7	1.391 (2)	C9—C17	1.542 (2)
N1—C18	1.465 (2)	C10—C11	1.381 (3)
N1—C5	1.467 (2)	C10—H10	0.9300
C1—C2	1.514 (3)	C11—C12	1.381 (3)
C1—C6	1.533 (2)	C11—H11	0.9300
C1—H1A	0.9700	C12—C13	1.388 (3)
C1—H1B	0.9700	C12—C15	1.512 (3)
C2—C3	1.511 (3)	C13—H13	0.9300
C2—H2A	0.9700	C14—H14A	0.9600
C2—H2B	0.9700	C14—H14B	0.9600
C3—C14	1.517 (3)	C14—H14C	0.9600
C3—C4	1.525 (2)	C15—H15A	0.9600
C3—H3	0.9800	C15—H15B	0.9600
C4—C5	1.530 (2)	C15—H15C	0.9600
C4—H4A	0.9700	C16—H16A	0.9600
C4—H4B	0.9700	C16—H16B	0.9600
C5—C6	1.532 (2)	C16—H16C	0.9600
C5—H5	0.9800	C17—H17A	0.9600
C6—C9	1.550 (2)	C17—H17B	0.9600
C6—H6	0.9800	C17—H17C	0.9600
C7—C10	1.404 (2)	C18—C19	1.469 (3)
C7—C8	1.407 (2)	C18—H18A	0.9700
C8—C13	1.392 (2)	C18—H18B	0.9700
C8—C9	1.528 (2)	C19—C20	1.168 (3)
C9—C16	1.539 (2)	C20—H20	0.9300
			
C7—N1—C18	117.18 (14)	C8—C9—C6	108.94 (13)
C7—N1—C5	121.04 (13)	C16—C9—C6	112.13 (14)
C18—N1—C5	117.14 (14)	C17—C9—C6	108.53 (13)
C2—C1—C6	112.89 (15)	C11—C10—C7	121.85 (16)
C2—C1—H1A	109.0	C11—C10—H10	119.1
C6—C1—H1A	109.0	C7—C10—H10	119.1
C2—C1—H1B	109.0	C10—C11—C12	121.23 (17)
C6—C1—H1B	109.0	C10—C11—H11	119.4
H1A—C1—H1B	107.8	C12—C11—H11	119.4
C3—C2—C1	111.02 (17)	C11—C12—C13	116.75 (17)
C3—C2—H2A	109.4	C11—C12—C15	121.55 (18)
C1—C2—H2A	109.4	C13—C12—C15	121.69 (18)
C3—C2—H2B	109.4	C12—C13—C8	124.06 (16)
C1—C2—H2B	109.4	C12—C13—H13	118.0
H2A—C2—H2B	108.0	C8—C13—H13	118.0
C2—C3—C14	112.77 (19)	C3—C14—H14A	109.5
C2—C3—C4	109.82 (15)	C3—C14—H14B	109.5
C14—C3—C4	111.95 (16)	H14A—C14—H14B	109.5
C2—C3—H3	107.3	C3—C14—H14C	109.5
C14—C3—H3	107.3	H14A—C14—H14C	109.5
C4—C3—H3	107.3	H14B—C14—H14C	109.5
C3—C4—C5	113.42 (14)	C12—C15—H15A	109.5
C3—C4—H4A	108.9	C12—C15—H15B	109.5
C5—C4—H4A	108.9	H15A—C15—H15B	109.5
C3—C4—H4B	108.9	C12—C15—H15C	109.5
C5—C4—H4B	108.9	H15A—C15—H15C	109.5
H4A—C4—H4B	107.7	H15B—C15—H15C	109.5
N1—C5—C4	110.99 (13)	C9—C16—H16A	109.5
N1—C5—C6	110.04 (13)	C9—C16—H16B	109.5
C4—C5—C6	109.98 (13)	H16A—C16—H16B	109.5
N1—C5—H5	108.6	C9—C16—H16C	109.5
C4—C5—H5	108.6	H16A—C16—H16C	109.5
C6—C5—H5	108.6	H16B—C16—H16C	109.5
C5—C6—C1	109.31 (14)	C9—C17—H17A	109.5
C5—C6—C9	113.23 (13)	C9—C17—H17B	109.5
C1—C6—C9	113.91 (13)	H17A—C17—H17B	109.5
C5—C6—H6	106.6	C9—C17—H17C	109.5
C1—C6—H6	106.6	H17A—C17—H17C	109.5
C9—C6—H6	106.6	H17B—C17—H17C	109.5
N1—C7—C10	120.02 (14)	N1—C18—C19	112.97 (15)
N1—C7—C8	122.17 (14)	N1—C18—H18A	109.0
C10—C7—C8	117.80 (15)	C19—C18—H18A	109.0
C13—C8—C7	118.29 (15)	N1—C18—H18B	109.0
C13—C8—C9	120.52 (14)	C19—C18—H18B	109.0
C7—C8—C9	121.15 (15)	H18A—C18—H18B	107.8
C8—C9—C16	108.30 (13)	C20—C19—C18	178.9 (2)
C8—C9—C17	110.19 (14)	C19—C20—H20	180.0
C16—C9—C17	108.76 (15)		
			
C6—C1—C2—C3	−57.2 (2)	C10—C7—C8—C9	178.42 (14)
C1—C2—C3—C14	−179.78 (16)	C13—C8—C9—C16	78.32 (19)
C1—C2—C3—C4	54.6 (2)	C7—C8—C9—C16	−99.15 (18)
C2—C3—C4—C5	−55.6 (2)	C13—C8—C9—C17	−40.5 (2)
C14—C3—C4—C5	178.33 (16)	C7—C8—C9—C17	142.00 (16)
C7—N1—C5—C4	−155.23 (15)	C13—C8—C9—C6	−159.48 (14)
C18—N1—C5—C4	49.6 (2)	C7—C8—C9—C6	23.05 (19)
C7—N1—C5—C6	−33.3 (2)	C5—C6—C9—C8	−48.98 (16)
C18—N1—C5—C6	171.56 (14)	C1—C6—C9—C8	−174.71 (13)
C3—C4—C5—N1	178.18 (14)	C5—C6—C9—C16	70.87 (17)
C3—C4—C5—C6	56.18 (18)	C1—C6—C9—C16	−54.85 (19)
N1—C5—C6—C1	−177.26 (14)	C5—C6—C9—C17	−168.97 (13)
C4—C5—C6—C1	−54.69 (17)	C1—C6—C9—C17	65.30 (18)
N1—C5—C6—C9	54.59 (17)	N1—C7—C10—C11	179.08 (18)
C4—C5—C6—C9	177.16 (12)	C8—C7—C10—C11	−1.8 (3)
C2—C1—C6—C5	56.8 (2)	C7—C10—C11—C12	1.3 (3)
C2—C1—C6—C9	−175.44 (15)	C10—C11—C12—C13	0.1 (3)
C18—N1—C7—C10	−18.0 (2)	C10—C11—C12—C15	−178.6 (2)
C5—N1—C7—C10	−173.17 (15)	C11—C12—C13—C8	−1.0 (3)
C18—N1—C7—C8	162.92 (16)	C15—C12—C13—C8	177.72 (19)
C5—N1—C7—C8	7.7 (2)	C7—C8—C13—C12	0.5 (3)
N1—C7—C8—C13	180.00 (15)	C9—C8—C13—C12	−177.06 (15)
C10—C7—C8—C13	0.9 (2)	C7—N1—C18—C19	86.5 (2)
N1—C7—C8—C9	−2.5 (2)	C5—N1—C18—C19	−117.36 (18)
