# Supplementary material for: Synthesis, crystal structure, Hirshfeld surface analysis and energy framework calculations of trans-3,7,9,9-tetra­methyl-10-(prop-2-yn-1-yl)-1,2,3,4,4a,9,9a,10-octa­hydro­acridine
Source: Acta Crystallogr E Crystallogr Commun. 2021 Feb 5;77(Pt 3):226–32. doi: 10.1107/S2056989021001183 (PMC8061106; doi:10.1107/S2056989021001183)
Supplement: Supplementary file 4 [file e-77-00226-sup4.pdf]

Supplementary Figures and Tables for "Synthesis, Crystal structure, Hirshfeld surface analysis and Energy frameworks calculations of the new *trans*-*N*-propargyl-3,7,9,9-tretramethyl-1,2,3,4,4a,9,9a,10-octahydroacridine"

Mauricio Acelas, Analio Dugarte-Dugarte, Arnold R. Romero Bohórquez, José Antonio Henao, José Miguel Delgado and Graciela Díaz de Delgado*.

Hirshfeld surface analysis.

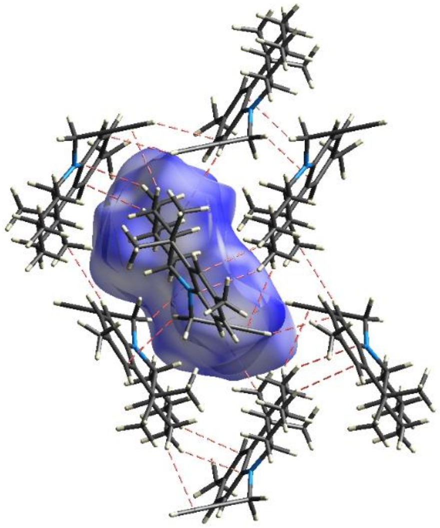

Figure S1. Neighboring molecules mapped over the Hirshfeld surface of a central molecule of compound **3**.

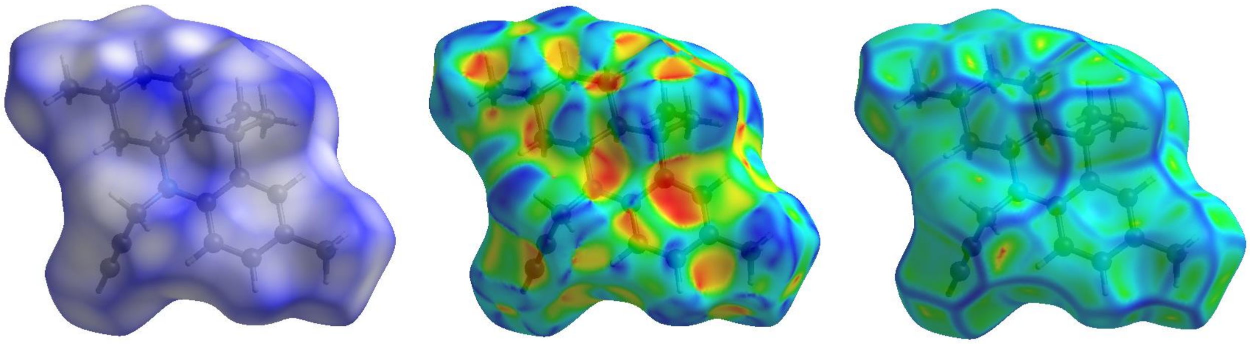

Figure S2. The parameters d_{norm}, shape index, and curveness mapped onto the Hirshfeld surface for compound **3**.

Geometry and energy optimization

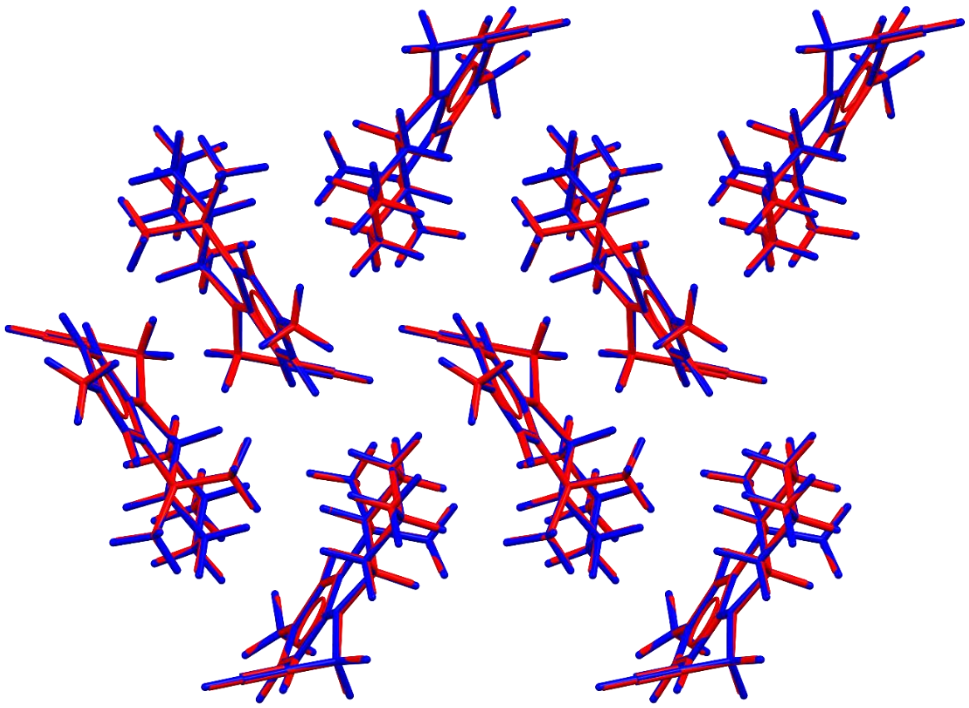

Figure S3. Superposition of the experimental (red) and the energy-minimized (blue) structure for compound **3**.

Table S1. Structural parameters from single-crystal X-ray diffraction (SC-XRD) and for PM7, PM6 and PM6-DH2 calculations for **3**. The calculations were carried out on a cluster with 16 molecules.

	Method						
	SC-XRD	PM7	Δ(Exp-PM7)	PM6	Δ(Exp-PM6)	PM6-DH2	Δ(Exp-PM6-DH2)
a	10.051	9.856	0.195	9.836	0.215	9.789	0.262
b	10.630	10.389	0.241	10.552	0.078	10.432	0.198
c	15.648	15.257	0.391	15.532	0.116	15.231	0.417
α	90	89.431	0.569	89.682	0.318	89.028	0.972
β	90	89.245	0.755	89.457	0.543	89.317	0.683
γ	90	89.431	0.569	91.679	1.679	91.762	1.762
V	1671.87	1561.96	109.910	1611.286	60.584	1554.327	117.543
% error-*V*	-	6.574		3.624		7.031	
d	1.118	1.197	0.079	1.16	0.042	1.203	0.085
% error-*d*	-	7.066		3.757		7.603	
RMSD	-	0.026		0.045		0.052	
$UME_{\mathbf{a,b,c}}$	-	0.572		0.136		0.691	
$UME_{\alpha,\beta,\gamma}$	-	0.631		0.847		1.139	
$UME_{\mathbf{a,b,c},\alpha,\beta,\gamma}$	-	0.453		0.491		0.716	
% error	-	1.468		1.073		1.822	

- For a description of the methods and parameters, see Korth *et al.*, 2010; Stewart, 2007, 2008, 2013, 2016, 2018.

Table S2. Structural parameters from SC-XRD and for PM7, PM6 and PM6-DH2 calculations for **3**. The calculations were carried out on a cluster with 8 molecules.

	Method						
	SC-XRD	PM7	Δ(Exp-PM7)	PM6	Δ(Exp-PM6)	PM6-DH2	Δ(Exp-PM6-DH2)
a	10.051	9.705	0.346	9.624	0.427	9.563	0.488
b	10.630	10.383	0.247	10.541	0.089	10.439	0.191
c	15.648	15.269	0.379	15.587	0.061	15.257	0.391
α	90	89.464	0.536	89.671	0.329	89.022	0.978
β	90	88.891	1.109	89.62	0.380	89.489	0.511
γ	90	89.67	0.330	93.89	3.890	93.625	3.625
V	1671.87	1538.143	133.727	1577.583	94.287	1519.831	152.039
% error-*V*	-	7.999		5.640		9.094	
d	1.118	1.215	0.097	1.185	0.067	1.23	0.112
% error-*d*	-	8.676		5.993		10.018	
RMSD	-	0.028		0.047		0.046	
$UME_{a,b,c}$	-	0.675		0.192		0.627	
$UME_{\alpha,\beta,\gamma}$	-	0.658		1.533		1.705	
$UME_{a,b,c,\alpha,\beta,\gamma}$	-	0.491		0.863		1.031	
% error	-	1.730		1.764		2.472	

- For a description of the methods and parameters, see Korth *et al.*, 2010; Stewart, 2007, 2008, 2013, 2016, 2018.

Table S3. Physicochemical parameters obtained from semi-empirical quantum chemistry calculations for compound **3**. The calculations were carried out on a cluster with 16 molecules.

	PM7	PM6	PM6-DH2
Molecular Formula, N° Atoms	$C_{320}H_{432}N_{16}$, 768	$C_{320}H_{432}N_{16}$, 768	$C_{320}H_{432}N_{16}$, 768
ΔH_f (KCal/mol)	201.467	464.876	64.687
Total Energy (KCal/mol)	-1109451.697	-1105437.146	-1105422.300
Energy of Atoms (KCal/mol)	1110233.475	1105903.296	1105903.296
Dispersion Energy (KCal/mol)	-580.30975	0	-414.763
H-bond Energy (KCal/mol)	0	0	0
MM Correction for >N (KCal/mol)	-	-1.274	-1.547
H.o.F. per unit cell (KCal)	12.592 (16 unit cells)	29.055 (16 unit cells)	4.043 (16 unit cells)
Ionization Potential (eV)	8.721	8.667	8.643
HOMO/LUMO Energies (eV)	-8.721/-0.561	-8.667/-0.428	-8.643/-0.432

Table S4. Physicochemical parameters associated with the semi-empirical quantum chemistry calculations for compound **3**. The calculations were carried out on a cluster with 8 molecules.

	PM7	PM6	PM6-DH2
Molecular Formula, N° Atoms	$C_{160}H_{216}N_{8}$, 384	$C_{160}H_{216}N_{8}$, 384	$C_{160}H_{216}N_{8}$, 384
ΔH_f (KCal/mol)	102.205	233.674	27.262
Total Energy (KCal/mol)	-554727.114	-552717.232	-552714.007
Energy of Atoms (KCal/mol)	555116.737	552951.648	552951.648
Dispersion Energy (KCal/mol)	-287.417	234.416	237.640
H-bond Energy (KCal/mol)	0	0	0
MM Correction for >N (KCal/mol)	-	-0.741	-0.813
H.o.F. per unit cell (KCal)	12.775 (8 unit cells)	29.209 (8 unit cells)	3.408 (8 unit cells)
Ionization Potential (eV)	8.735	8.649	8.654
HOMO/LUMO Energies (eV)	-8.736/-0.568	-8.650 -0.408	-8.654/-0.445

Table S5. Comparison of selected parameters of the experimental crystal structure (SC-XRD) and the PM7 calculations for compound **3**.

	Method	
	SC-XRD	PM7
a (Å)	10.051	9.856
b (Å)	10.630	10.389
c (Å)	15.648	15.257
α (°)	90	89.431
β (°)	90	89.245
γ (°)	90	89.431
V (Å^3)	1671.87	1561.96
d (g/cm^3)	1.118	1.197
$UME_{\mathbf{a,b,c},\alpha,\beta,\gamma}$	0.453	
RMSD	0.026	

*UME, *Unsigned Mean Error*, $UME_i = \frac{1}{n}\sum_{j=1}^{n}\left|R_{ij}^{\exp\ or\ CSD} - R_{ij}^{Calc}\right|$

Table S6. Energetic parameters calculated for compound **3**.

Molecular Formula, - N° Atoms	$C_{320}H_{432}N_{16}$, 768
ΔH_f (KCal/mol)	201.467
Total Energy (KCal/mol)	-1109451.697
Energy of Atoms (KCal/mol)	1110233.475
Dispersion Energy (KCal/mol)	-580.30975
H-bond Energy (KCal/mol)	0
MM Correction for >N (KCal/mol)	-
H.o.F. per unit cell (KCal)	12.592 (16 unit cells)
Ionization Potential (eV)	8.721
HOMO/LUMO Energies (eV)	-8.721/-0.561

References:

Korth, M., Pitoňák, M., Řezáč, J. & Hobza, P. (2010). *J. Chem. Theory Comput.* **6**, 344-352.

Stewart, J. J. P. (2007). *J. Mol. Model.* **13**, 1173-1213.

Stewart, J. J. P. (2008). *J. Mol. Model.* **14**, 499-535.

Stewart, J. J. P. (2013). *J. Mol. Model.* **19**, 1-32.

Stewart, J. J. P. (2016). *MOPAC2016.* Version 18.258W. Stewart Computational Chemistry: Colorado Springs, CO, USA.

Stewart, J. J. P. (2018). *MAKPOL.* Stewart Computational Chemistry: Colorado Springs, CO, USA.
